# Resurgence of *Bordetella pertussis*, including one macrolide-resistant isolate, France, 2024

**DOI:** 10.2807/1560-7917.ES.2024.29.31.2400459

**Published:** 2024-08-01

**Authors:** Carla Rodrigues, Valérie Bouchez, Anaïs Soares, Sabine Trombert-Paolantoni, Fatima Aït El Belghiti, Jérémie F Cohen, Nathalie Armatys, Annie Landier, Thomas Blanchot, Marie Hervo, REMICOQ study group, Julie Toubiana, Sylvain Brisse, REMICOQ study group, Nathalie Brieu, Jenny Gallou, Audrey Homor, Morgane Choquet, Marie Kempf, Hélène Pailhoriès, Carine Dumollard, Dominique De Briel, Margaux Allain, Guilène Barnaud, Luce Landraud, Julien Bador, Gaëlle Cuzon, Benjamin Aubry, Jean Thomin, Ghislaine Descours, Ani Horikian, Anne-Lise Maucotel, Hélène Salord, Aubin Souche, Christelle Jost, Frederic Queuche, Agnès Ferroni, Chloé Plouzeau, Stéphane Bland, Hélène Petiprez, Marie Sarah Fangous, Florence Le Gall, Anne Vachee, Caroline Piau

**Affiliations:** 1 Institut Pasteur, Université Paris Cité, Biodiversity and Epidemiology of Bacterial Pathogens, Paris, France; 2 National Reference Center for Whooping Cough and other Bordetella infections, Institut Pasteur, Paris, France; 3 Laboratoire Eurofins Biomnis, Lyon, France; 4 Laboratoire Cerba, Saint Ouen l’Aumône, France; 5 Santé publique France, Infectious Diseases Department, The French Public Health Agency, Saint-Maurice, France; 6 Department of General Paediatrics and Paediatric Infectious Diseases, Université Paris Cité, Hôpital Necker-Enfants Malades, APHP, Paris, France; 7 Centre for Research in Epidemiology and Statistics (Inserm UMR 1153), Université Paris Cité, Paris, France; 8 The members of the REMICOQ study group are listed under Collaborators; * These authors contributed equally to this work and share first authorship.; ** These authors co-supervised the work and share last authorship.

**Keywords:** *Bordetella pertussis*, post-covid-19 resurgence, pertactin production, FIM2 fimbriae, macrolide resistance

## Abstract

As other European countries, France is experiencing a resurgence of pertussis in 2024. Between 1 January and 31 May 2024, 5,616 (24.9%) positive *Bordetella pertussis* qPCR tests were identified, following a 3-year period of almost null incidence. Of 67 cultured and whole genome sequenced *B. pertussis* isolates, 66 produced pertactin and 56 produced FIM2, in contrast to pre-COVID-19 years. One isolate of genotype Bp-AgST4 was resistant to macrolides. Pertussis resurgence may favour isolates that produce FIM2 and pertactin.

*Bordetella pertussis* (*Bp*) is the main agent of whooping cough or pertussis, which can be fatal for at-risk people, particularly for infants too young to be vaccinated and whose mothers were not vaccinated during pregnancy [1]. Here, we report on the ongoing resurgence of pertussis in France and describe the microbiological characteristics of the associated *Bp* population.

## Outpatient laboratory surveillance by PCR

**Figure 1:**
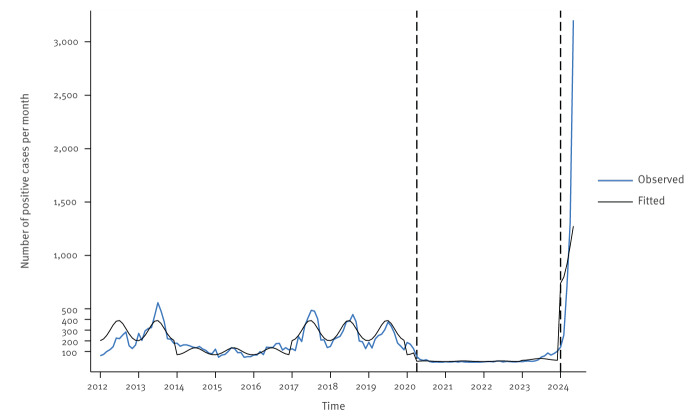
Time series analysis of positive qPCR tests targeting IS*481,* France, 2012–2024

The two largest French outpatient laboratories conduct more than 90% of the ambulatory diagnostic tests for *Bp* in France, providing a representative overview of its nation-wide circulation. A pertussis case was defined as a person with a positive result in a real-time PCR (qPCR) targeting IS*481* from nasopharyngeal swabs. Based on a quasi-experimental interrupted time series analysis relying on negative binomial regression models, we analysed the dynamics of pertussis cases over time (Figure 1). Details of the statistical analyses are appended in the Supplementary text 1. 

The black line reflects the values predicted by the negative binomial regression model. On the x-axis, the year marks correspond to the beginning of the year (January). Note that the data for 2012 were provided by only one of the two laboratories. The first vertical dashed line indicates the beginning of the first COVID-19 lockdown (1 April 2020). The second vertical dashed line corresponds to the start of the current outbreak and subsequent national alert (1 January 2024).

Before 1 April 2020 (first COVID-19 lockdown), epidemic cycles were observed, with the last peak in 2017/19 [2]. Between 1 April 2020 and 31 December 2023, the average number of monthly *Bp* cases was 16 (standard deviation (SD): 26). After 1 January 2024, the average number was much higher (mean = 1,123; SD: 1,250), reaching 3,202 cases in May. The median age of the cases in 2024 was 17.7 years (interquartile range (IQR): 8.4–42.9), including 19% 0–5-year-olds, 32% 6–17-year-olds and 49% ≥ 18-year-olds. The uninterrupted time-series model identified a statistically significant increase in pertussis cases after January 2024, when compared with the period before the COVID-19 pandemic, corresponding to an adjusted incidence rate ratio (aIRR) of 3.66 (95% confidence interval: 1.42–9.39) (Table).

**Table:** Pertussis incidence before, during and after the COVID-19 pandemic: interrupted time-series analysis, France, 2012–2024 (n = 26,060)

	Adjusted IRR^a^	95% CI	p value
Overall number of positive PCRs (n = 26,060)			
Pre-COVID-19	Reference		
COVID-19 pandemic^b^	0.09	0.06–0.13	< 0.001
Post-COVID-19	3.66	1.42–9.39	0.007
Number of positive PCRs, by age^c^			
0–5 years (n = 5,716)			
Pre-COVID-19	Reference		
COVID-19 pandemic^b^	0.13	0.08–0.18	< 0.001
Post-COVID-19	3.29	1.28–8.48	0.014
6–17 years (n = 6,717)			
Pre-COVID-19	Reference		
COVID-19 pandemic^b^	0.09	0.0–0.13	< 0.001
Post-COVID-19	4.58	1.79–11.76	0.002
≥ 18 years (n = 13,430)			
Pre-COVID-19	Reference		
COVID-19 pandemic^b^	0.08	0.05–0.11	< 0.001
Post-COVID-19	3.62	1.41–9.29	0.008
Proportion of positive PCRs			
Pre-COVID-19	Reference		
COVID-19 pandemic^b^	0.17	0.02–1.49	0.110
Post-COVID-19	1.86	0.19–17.96	0.591
Sensitivity analysis^d^			
Pre-COVID-19	Reference		
COVID-19 pandemic^b^	0.09	0.06–0.13	< 0.001
Post-COVID-19	3.52	1.34–9.27	0.011

CI: confidence interval; IRR: incidence rate ratio.

^a^ Negative binomial model adjusted on seasonality and 3-year pertussis cycles.

^b^ Between April 2020 and December 2023.

^c^ Age data were missing for 197 cases (less than 1%).

^d^ Seasonality-adjusted with calendar month instead of sine/cosine terms.

## *Bordetella pertussis* isolates

*Bordetella* isolates are collected through the REMICOQ network, which comprises laboratories from the RENACOQ paediatric hospital-based surveillance network [3] and additional collaborative hospital and outpatient laboratories. The qPCR-positive samples referred to the National Reference Centre (NRC) are used to attempt culture and isolation of the strain. *Bordetella pertussis* isolates were analysed at the NRC for species identification by MALDI-TOF and for haemolysis, oxidase, urease and qPCR targeting IS*481* and *ptxP* or *BP3385* [4], if necessary. The in-vitro antigen production was analysed by Western blot for pertactin [5], and by agglutination assays for fimbrial proteins. Antibiotic susceptibility testing was performed by disk diffusion for ampicillin, cephalexin, streptomycin, trimethoprim-sulfamethoxazole, erythromycin, azithromycin, clarithromycin and spiramycin. Macrolide resistance was confirmed by E-test.

**Figure 2:**
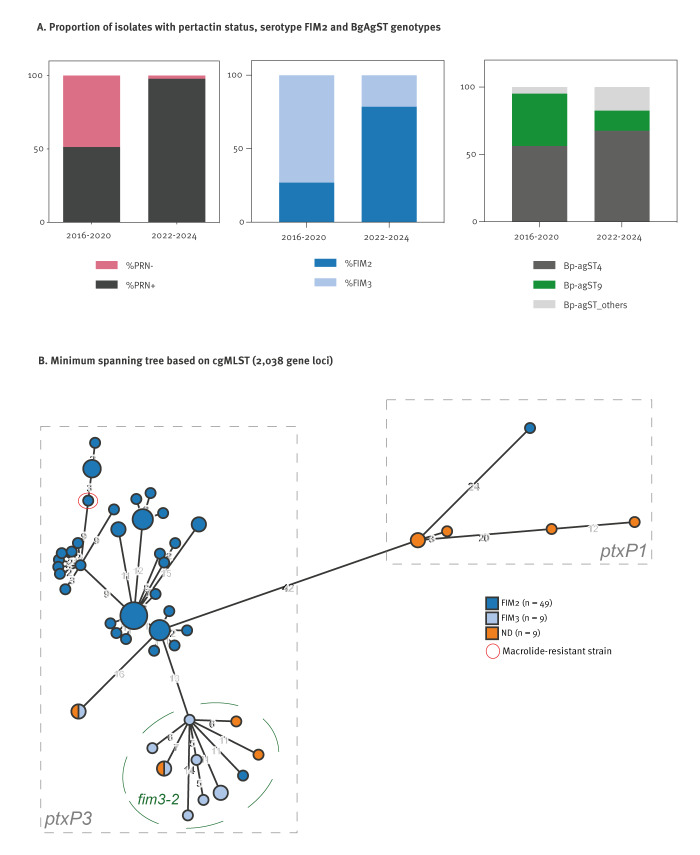
Characteristics of *Bordetella pertussis* isolates from France, June 2023–April 2024 (n = 67) vs 2016–2020

Bp-agST: *Bordetella pertussis* antigen sequence type; cgMLST: core genome multilocus sequence typing; FIM: fimbriae; ND: not determined; PRN: pertactin; PtxP: pertussis toxin promoter.

The NRC analysed nine *Bp* isolates from June to December 2023 and 58 isolates from January to May 2024; all were haemolytic on Bordet–Gengou agar as expected. The median age of all patients was 0.3 years (IQR: 0.2–7.5 years), and 30 of 39 patients for whom hospitalisation status was known were hospitalised (18 of them were younger than 2 months). Of the total 67 isolates, 66 produced pertactin, in contrast to *Bp* isolates from France in pre-COVID-19 pandemic years when 117 (51.3%) of 228 isolates collected from 2016 to 2020 were pertactin-negative [4]. Among the 67 recent isolates, 56 produced FIM2 (Figure 2A), whereas FIM3-producing isolates were predominant before 2020 [4]. All isolates from 2024 produced pertussis toxin and filamentous haemagglutinin, similar to virtually all pre-pandemic *Bp* isolates. One isolate (FR7302, February 2024) was resistant to macrolides (MIC > 256 mg/L for azithromycin, clarithromycin and erythromycin) but remained susceptible to the other antibiotics tested, except for cephalexin (intrinsic resistance). This isolate had a previously described macrolide resistance mutation in the 23S rRNA gene sequence [6]. It was collected from a man in his late 70s and was pertactin-negative.

Panel B: The numbers on the branches indicate the number of allelic differences among cgST profiles. Each circle represents a cgST type, coloured by FIM serotype. The diameter of circles is related to the number of isolates they comprise; the largest circle comprises seven isolates.

## Genomic diversity of *Bordetella pertussis* isolates

Genotyping analyses based on genomic sequences (PRJEB42353) were conducted as previously described [7,8]. The data showed 47 distinct core-genome sequence types (cgST) genotypes; four of them were isolated 2–7 times (Figure 2B).

Six isolates had the ancestral genotype of the promoter sequence of the pertussis toxin gene cluster, *ptx*P1, whereas 61 had the evolved genotype *ptx*P3. Most of the latter had the *fim3–1* allele (n = 49) of the type 3 fimbriae gene, whereas only 12 isolates had the *fim3–2* allele. The macrolide-resistant isolate was *ptxP3* and *fim3–1* and produced FIM2 (Figure 2B). This isolate was phylogenetic closely related to macrolide-resistant isolates from China but also to macrolide-susceptible isolates from France in 2024; For details on the phylogenetic diversity of macrolide-resistant isolates, we refer to the appended material in the Supplementary text 2 and Figure S1.

## Discussion

The epidemiology of pertussis follows a cyclical pattern, with epidemic periods every 3–5 years [9]. In France the two last epidemic peaks were observed in 2012/13 and in 2017/18 [2,4]. During the COVID-19 pandemic, the circulation of *Bp* was strongly reduced in France [2] and other European countries [10]. In 2022, a transient increase was observed for *Bordetella parapertussis,* the second agent of whooping cough, but not for *Bp* [11]. Here, we report a sharp and currently ongoing increase in the number of pertussis cases in France, with an important accentuation since March 2024. Following a long period with almost no reported pertussis cases, the sharp increase in cases has become significant since January 2024. The incidence in 2024 was even higher than in the pre-COVID-19 pandemic period, even when considering the cyclical pattern and seasonality of pertussis. A similar resurgence of other common airborne infectious diseases has been observed with relaxed physical distancing measures, such as for respiratory syncytial virus and invasive group A streptococci [12].

It is possible that highly reduced exposure to viral and bacterial pathogens for several years reduced the boosting of population-level immunity by natural (often asymptomatic) infections. Since 2013, the French pertussis vaccine schedule has comprised two primary doses at 2 and 4 months of age, a first booster at 11 months of age, and additional boosters at 6, 11–13 and 25 years. There is no evidence of a reduction in pertussis vaccination coverage in France, even though a delay was observed during the early phase of the pandemic. 

Another possible driver of the intense resurgence reported here, specific to pertussis, is that *Bp* population evolution, mostly by loss of pertactin expression [13,14], may have impacted the effectiveness of current vaccines. However, even though few isolates were obtained compared with the qPCR-positive tests, the fact that all except one of the sampled 2024 isolates were pertactin-positive is not in favour of this hypothesis. In most high-income countries, vaccination involves acellular vaccines containing two to five antigens: pertussis toxin and filamentous haemagglutinin, and sometimes in addition pertactin and the fimbriae proteins FIM2 and FIM3. In France, one vaccine frequently used for the primary schedule in infants, and some booster vaccines do not contain pertactin, which may have created an opportunity for pertactin-positive *Bp* isolates to spread more efficiently. We also show that FIM2 fimbriae expression is predominant, whereas nearly two thirds of *Bp* isolates before the COVID-19 pandemic expressed FIM3 [4], as in other countries [15,16].

The large-scale genomic evolution of *Bp* has been marked by key mutations, including in the promoter of the pertussis toxin gene cluster (*ptx*P) or the *fim3* gene [17,18]. In most high-income countries, *ptx*P3 isolates have largely replaced the ancestral *ptx*P1 genotype. Here, we observed that *ptxP1* isolates were not uncommon (representing 11%), whereas only three (1%) *ptxP1* had been collected in France between January 2016 and April 2020. The *ptx*P1 isolates were predominant before the emergence of *ptx*P3 ones in the 1980s but became a minority after the introduction of acellular vaccines [17,18]. The two alleles of gene *fim3* coding for FIM3 fimbriae, *fim3–1* and *fim3–2*, that divide the *ptxP3* phylogenetic branch of *Bp*, were both commonly observed in the pre-COVID-19 pandemic era.

Until now, macrolide resistance has seldom been reported, with the notable exception of Asia and more particularly China, where such isolates have become highly prevalent in the last years [19]. Only one *Bp* isolate had previously been reported in France (and in Europe) in 2011 [6]. The *Bp* isolates resistant to macrolides that were described previously belong to different genotypes; In the appended Supplementary Figure S1, we provide their phylogenetic diversity: Bp-AgST37 found in China (characterised by *ptx*P1 and *fhaB*3 alleles), Bp-AgST8 (*ptx*P1 and *fhaB*1) found for isolate A228 from the United States (US) in 1994 and Bp-AgST4 (*ptx*P3 and *fhaB*1), which was observed for strain ATCC BAA1335 collected in 1999 in the US and more recently for isolates from China, also classified as ‘MT28’ based on multilocus variable number tandem repeats analysis (MLVA). The macrolide-resistant isolate collected in 2024 in France (FR7302) was of genotype Bp-AgST4 and thus genotypically related to isolates from China. In fact, our phylogenetic analysis indicates that the closest relatives of FR7302 are isolates from China (group *ptxP3*-MRBP2). Although these genetic data may suggest importation from China, no epidemiological link was found. Besides, closely related macrolide-susceptible Bp-AgST4 isolates circulate in France, as also evident in the appended Supplementary Figure S1. Therefore, an alternative possibility is the evolution towards macrolide resistance in France from a Bp-AgST4 macrolide-susceptible lineage. So far, macrolide resistance remains rare in France. It is possible that it incurs a high fitness cost to the bacteria, preventing the rapid spread of this concerning phenotype [20]. The use of macrolide antimicrobials should remain prudent to avoid creating a selective advantage for macrolide-resistant strains.

## Conclusions

We report a sharp increase in pertussis cases in France in 2024, nearly simultaneous with similar increases in other European countries such as Czechia, Denmark and Spain. The main risk is for non- or partially immunised infants younger than 6 months, who represent the group with the highest morbidity and mortality from pertussis, underscoring the critical importance of vaccinating pregnant women against pertussis to protect young infants. In addition, the report of one macrolide-resistant *B. pertussis* isolate in a European country calls for reinforced surveillance.

## REMICOQ study group

**Nathalie Brieu, Jenny Gallou, Audrey Homor** (Centre Hospitalier du Pays d’Aix, Aix-en-Provence, France)**; Morgane Choquet** (Department of Bacteriology, Amiens University Hospital, Amiens, France); **Marie Kempf, Hélène Pailhoriès** (Centre Hospitalier Universitaire d'Angers, Bacteriology Department, Angers, France); **Carine Dumollard** (Centre Hospitalier Métropole Savoie; Laboratory of Medical Biology, Chambéry, France); **Dominique De Briel** (Hopitaux Civils de Colmar, Colmar, France) ; **Margaux Allain, Guilène Barnaud, Luce Landraud** (Microbiology and Hygiene Service, Hôpital Louis-Mourier, Colombes, France) ; **Julien Bador** (Centre Hospitalier Universitaire Dijon Bourgogne, Dijon, France); **Gaëlle Cuzon** (AP-HP Hôpital Bicêtre, Le Kremlin-Bicêtre, France) ; **Benjamin Aubry, Jean Thomin** (Centre Hospitalier du Mans, Le Mans, France) ; **Ghislaine Descours**, **Ani Horikian, Anne-Lise Maucotel**, **Hélène Salord, Aubin Souche** (Hôpital de la Croix Rousse, Centre de Biologie Nord, Institut des Agents Infectieux, Lyon, France) ; **Christelle Jost** (Hôpital Mercy CHR Metz Thionville, Metz, France) ; **Frederic Queuche** (Centre Hospitalier de Montluçon - Néris les Bains, Montluçon, France) ; **Agnès Ferroni** (AP-HP Hôpital Necker-Enfants Malades, Paris, France) ; **Chloé Plouzeau** (Centre Hospitalier Universitaire de Poitiers) ; **Stéphane Bland, Hélène Petiprez** (Centre Hospitalier Annecy Genevois – site d’Annecy, Pringy, France) ; **Marie Sarah Fangous, Florence Le Gall** (Centre Hospitalier de Cornouaille Quimper Concarneau, Quimper, France) ; **Anne Vachee** (Centre Hospitalier de Roubaix, Roubaix, France) ; **Caroline Piau** (Centre Hospitalier Universitaire Rennes Pontchaillou, Rennes, France).

## Supplementary Data

395000 Supplement
