# Supplementary material for: Resurgence of Bordetella pertussis, including one macrolide-resistant isolate, France, 2024
Source: Euro Surveill. 2024 Aug 1;29(31):2400459. doi: 10.2807/1560-7917.ES.2024.29.31.2400459 (PMC11295439; doi:10.2807/1560-7917.ES.2024.29.31.2400459)
Supplement: Supplement [file 24-00459_BRISSE_Supplement.pdf]

Supplementary Appendix

Resurgence of *Bordetella pertussis*, including one macrolide-resistant isolate, France, 2024

Carla Rodrigues1,2*, Valérie Bouchez1,2*, Anaïs Soares3, Sabine Trombert-Paolantoni4, Fatima Aït El Belghiti5, Jérémie F Cohen6,7, Nathalie Armatys1,2, Annie Landier1,2, Thomas Blanchot3, Marie Hervo3, REMICOQ study group8, Julie Toubiana1,2,6, Sylvain Brisse1,2

1. Institut Pasteur, Université Paris Cité, Biodiversity and Epidemiology of Bacterial Pathogens, Paris, France
2. National Reference Center for Whooping Cough and other Bordetella infections, Institut Pasteur, Paris, France
3. Laboratoire Eurofins Biomnis, Lyon, France
4. Laboratoire Cerba, Saint Ouen l'Aumône, France
5. Santé publique France, Infectious Diseases Department, The French Public Health Agency, Saint-Maurice, France
6. Department of General Paediatrics and Paediatric Infectious Diseases, Université Paris Cité, Hôpital Necker-Enfants Malades, APHP, Paris, France
7. Centre for Research in Epidemiology and Statistics (Inserm UMR 1153), Université Paris Cité, Paris, France
8. The members of the REMICOQ study group are listed under Collaborators

* These authors contributed equally to this work and share first authorship.

Correspondence: Julie Toubiana (julie.toubiana@pasteur.fr), Sylvain Brisse (sbrisse@pasteur.fr)

Disclaimer :

"This supplementary material is hosted by Eurosurveillance as supporting information alongside the article Resurgence of Bordetella pertussis, including one macrolide-resistant isolate, France, 2024, on behalf of the authors, who remain responsible for the accuracy and appropriateness of the content. The same standards for ethics, copyright, attributions and permissions as for the article apply. Supplements are not edited by Eurosurveillance and the journal is not responsible for the maintenance of any links or email addresses provided therein."

Supplementary text 1: Statistical Analyses

We conducted a quasi-experimental interrupted time series analysis relying on negative binomial regression models, with a time unit of 1 month, to analyze the changes in the incidence of positive Bordetella PCRs over time. All positive PCR results were included in the models. The models accounted for seasonality (using one pair of sine/cosine terms), a binary variable to define periods before and after the COVID-19 pandemic (this variable was coded 0 before April 2020, 1 between April 2020 and December 2023, and 2 starting in January 2024) and overdispersion of data. The models also included a dummy variable (with 36-month periods) to adjust for long-term cycles commonly observed in pertussis epidemiology. In such models, an incidence rate ratio (IRR) greater than 1 indicates that the corresponding variable is associated with an increase in pertussis incidence. We also conducted a sensitivity analysis in which seasonality was adjusted using calendar month. We used Stata/SE 18.0 (StataCorp LP, College Station, TX, United States) for all analyses.

Supplementary text 2: genomic analysis of international macrolide-resistant *B. pertussis*

A phylogenetic analysis was performed focusing on *B. pertussis* isolates resistant to macrolides (MRBP), based on the dataset described in Bridel *et al*. 2022 (1), supplemented with genomes from Wu *et al*. (2) downloaded from NCBI, and adding all French isolates from 2024 (n=186).

Based on this dataset, four different genotypes of MRBP isolates were identified:

- ***ptxP1*-MRBP1** clade grouped macrolide resistant isolates originating mostly from China, with 2 genomes from Japan (3) and 2 from the USA. All were characterized by a Bp-AgST37 (characterized by a *ptxP1* and *fhaB*3 genotype), as defined in Bridel *et al*. 2022 (1).
- one ***ptxP1*-MRBP2** clade contained only one macrolide-resistant isolate A228 collected in 1994 in the USA (4) in Arizona. This isolate was Bp-AgST8, characterized by *ptxP1* and *fhaB1* alleles.
- the clade ***ptxP3*-MRBP1** contained the macrolide resistant isolate ATCC BAA1335 collected in 1999 in the USA (5), with genotype Bp-AgST4, characterized by a *ptxP3* and *fhaB1* alleles.
- the clade ***ptxP3*-MRBP2** contained 5 macrolide resistant isolates: 1 from France collected in 2024 (FR7302) and 4 from China (P745 and Bp20, BP0066 and GZBP22005). All were Bp-AgST4, with *ptxP*3, *fhaB*1 and *fim3*-1 alleles. The MRBP isolate collected in France in 2024 (FR7302) was found to be phylogenetically associated with *ptxP*3-MRBP3 P745 isolate from Shanghai

(2022) characterized by a MT28 MLVA profile (2). Only 2 cgMLST loci differed between these two isolates. These two isolates were also closely related to 2 other macrolide resistant isolates, BP0066 and GZBP22005, both collected in China but also to another MT28 isolate from China (P20), susceptible to macrolides (2), indicating that co-existence of both phenotypes (susceptible & resistant) in the same *ptxP3*-MRBP2 genetic clade as previously reported (2).

- the last clade, ***ptxP3*-MRBP3,** contained 7 isolates collected in France in 2011: 6 isolates (2 susceptible and 4 resistant) from the same patient (6) and another isolate, FR4808, collected the same year from another patient but susceptible to macrolides. All were characterized by genotype Bp-AgST68 with *ptxP*21, *fhaB*1and *fim3*-2 alleles.

<u>**Supplementary Figure 1**</u>

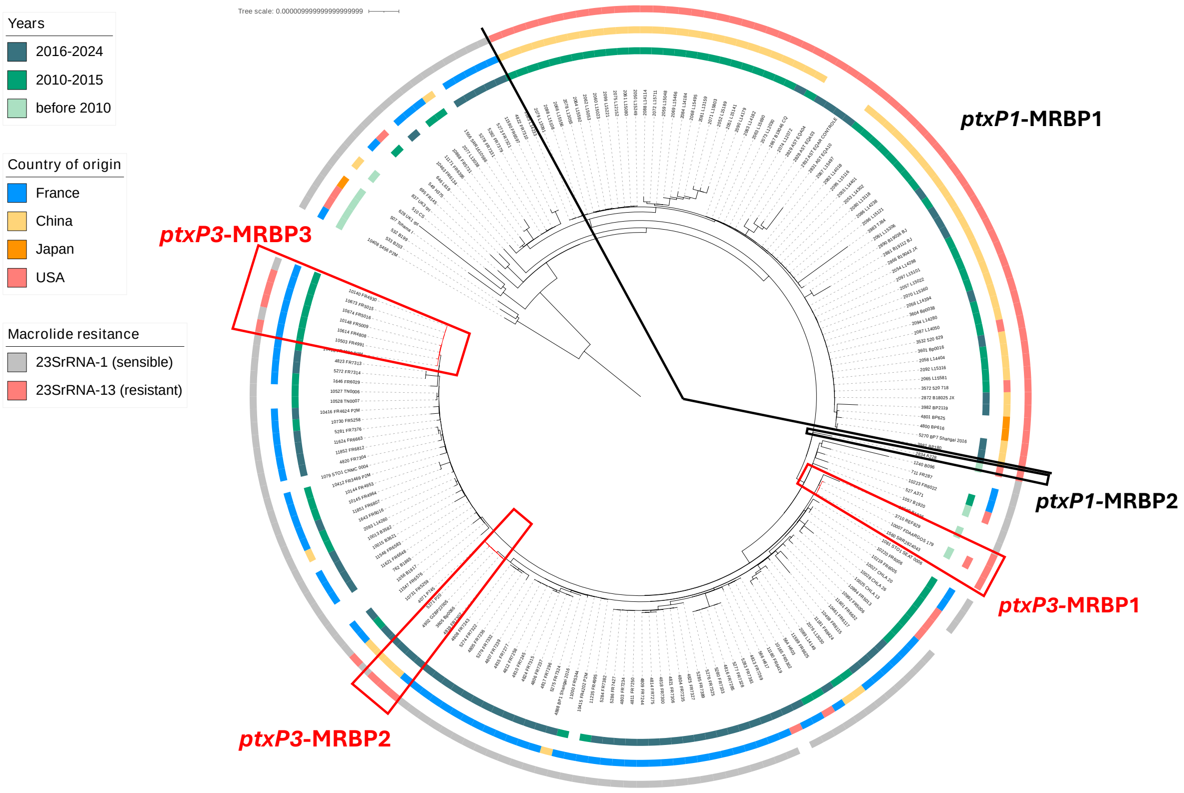

Phylogenetic tree of *B. pertussis* isolates from France, 2024, compared to isolates from Bridel *et al.*, 2022 (1) and Wu *et al.*, 2022 (2).

The tree was constructed from the concatenation of the sequence alignments of 2 032 cgMLST genes. First (internal) circle: years of collection (light green: before 2010, intermediate green: 2010-2015; dark green: 2016-2024). Second circle: Country of origin (blue: France, light orange: China, dark orange: Japan, red: USA). External circle: macrolide resistance genotype (grey: susceptible [allele 23SrRNA-1]; red: resistant [allele 23SrRNA-13]). The different groups of macrolide-resistant Bp (MRBP) isolates are indicated as black (*ptxP1*) and red (*ptxP3*) sectors (*ptxP1*-MRBP1, *ptxP1*-MRBP2, *ptxP3*-MRBP1, *ptxP3*-MRBP2 and *ptxP3*-MRBP3).

Supplementary references

1. Bridel S, Bouchez V, Brancotte B, Hauck S, Armatys N, Landier A, et al. A comprehensive resource for Bordetella genomic epidemiology and biodiversity studies. *Nat Commun*. 2022;13(1):3807
2. Wu X, Du Q, Li D, Yuan L, Meng Q, Fu Z, Xu H, Yao K, Zhao R. A Cross-Sectional Study Revealing the Emergence of Erythromycin-Resistant *Bordetella pertussis* Carrying *ptxP3* Alleles in China. Front Microbiol. 2022;13:901617.
3. Koide K, Uchitani Y, Yamaguchi T, Otsuka N, Goto M, Kenri T, Kamachi K. Whole-genome comparison of two same-genotype macrolide-resistant Bordetella pertussis isolates collected in Japan. PLoS One. 2024;19(2):e0298147.
4. Lewis K, Saubolle MA, Tenover FC, Rudinsky MF, Barbour SD, Cherry JD. Pertussis Caused by an Erythromycin-Resistant Strain of Bordetella Pertussis. *Pediatr Infect Dis. J.* 1995;14:388–39126
5. Bartkus JM, Juni BA, Ehresmann K, Miller CA, Sanden GN, Cassiday PK, Saubolle M, Lee B, Long J, Harrison AR Jr, Besser JM. Identification of a mutation associated with erythromycin resistance in Bordetella pertussis: implications for surveillance of antimicrobial resistance. J Clin Microbiol. 2003;41(3):1167-72.
6. Guillot S, Descours G, Gillet Y, Etienne J, Floret D, Guiso N. Macrolide-resistant Bordetella pertussis infection in newborn girl, France. *Emerg Infect Dis.* 2012;18(6):966–8.
